# Intraoperative neural signals predict rapid antidepressant effects of deep brain stimulation

**DOI:** 10.1038/s41398-021-01669-0

**Published:** 2021-11-03

**Authors:** Mohammad S. E. Sendi, Allison C. Waters, Vineet Tiruvadi, Patricio Riva-Posse, Andrea Crowell, Faical Isbaine, John T. Gale, Ki Sueng Choi, Robert E. Gross, Helen S. Mayberg, Babak Mahmoudi

**Affiliations:** 1 Wallace H. Coulter Department of Biomedical Engineering at Georgia Institute of Technology, Emory University, Atlanta, GA USA; 2 Department of Electrical and Computer Engineering, Georgia Institute of Technology, Atlanta, GA USA; 3 Departments of Psychiatry & Neuroscience, Center for Advanced Circuit Therapeutics, Icahn School of Medicine at Mount Sinai, New York, NY USA; 4 Department of Psychiatry and Behavioral Sciences, Emory University School of Medicine, Atlanta, GA USA; 5 Department of Neurosurgery, Emory University School of Medicine, GA, Atlanta, GA USA; 6 Departments of Radiology & Neurosurgery, Center for Advanced Circuit Therapeutics, Icahn School of Medicine at Mount Sinai, New York, NY USA; 7 Departments of Neurology, Neurosurgery, Psychiatry and Neuroscience, Center for Advanced Circuit Therapeutics, Icahn School of Medicine at Mount Sinai, New York, NY USA; 8 Department of Biomedical Informatics, Emory University School of Medicine, Atlanta, GA USA

**Keywords:** Predictive markers, Neuroscience, Depression

## Abstract

Deep brain stimulation (DBS) of the subcallosal cingulate (SCC) is a promising intervention for treatment-resistant depression (TRD). Despite the failure of a clinical trial, multiple case series have described encouraging results, especially with the introduction of improved surgical protocols. Recent evidence further suggests that tractography targeting and intraoperative exposure to stimulation enhances early antidepressant effects that further evolve with ongoing chronic DBS. Accelerating treatment gains is critical to the care of this at-risk population, and identification of intraoperative electrophysiological biomarkers of early antidepressant effects will help guide future treatment protocols. Eight patients underwent intraoperative electrophysiological recording when bilateral DBS leads were implanted in the SCC using a connectomic approach at the site previously shown to optimize 6-month treatment outcomes. A machine learning classification method was used to discriminate between intracranial local field potentials (LFPs) recorded at baseline (stimulation-naïve) and after the first exposure to SCC DBS during surgical procedures. Spectral inputs (theta, 4–8 Hz; alpha, 9–12 Hz; beta, 13–30 Hz) to the model were then evaluated for importance to classifier success and tested as predictors of the antidepressant response. A decline in depression scores by 45.6% was observed after 1 week and this early antidepressant response correlated with a decrease in SCC LFP beta power, which most contributed to classifier success. Intraoperative exposure to therapeutic stimulation may result in an acute decrease in symptoms of depression following SCC DBS surgery. The correlation of symptom improvement with an intraoperative reduction in SCC beta power suggests this electrophysiological finding as a biomarker for treatment optimization.

## Introduction

The long-term efficacy of deep brain stimulation (DBS) to the subcallosal cingulate (SCC) in patients with treatment-resistant depression (TRD) has been repeatedly demonstrated in independent, open-label studies with additional evidence of sustained response and remission over years of ongoing treatment [1–5]. While these accounts have documented a sustained effectiveness of this treatment with chronic stimulation, there are differences in the timeline of recovery across patients. This may have been one of the factors explaining the failure of the industry-sponsored trial to meet its primary endpoint at 6 months, when a considerable number of patients in the open-label phase responded favorably after 18 months in the study [6].

An understanding of the factors influencing the time-course of response to DBS and identifying early predictors of response will be key to future study designs. While effects of DBS are conventionally discussed on the scale of several months, a recent report demonstrated a pronounced and consistent rapid antidepressant response to SCC DBS [7]. Specifically, the magnitude of antidepressant effects observed within a week of surgery was enhanced with intraoperative exposure to bilateral stimulation at the tractography-defined “optimal” target. Authors speculate that the addition of a longer exposure to intraoperative stimulation may have accounted for an enhancement of an acute antidepressant effect of DBS. These clinical observations have not yet been understood mechanistically.

Intraoperative electrophysiological recordings could illuminate the brain changes that correlate with the clinical early treatment gains. In the absence of known electrophysiological predictors of an early antidepressant response to DBS, we cast hypothesis testing broadly as a classification problem. Moreover, we selected an interpretable machine learning approach [8], such that classification models can be interrogated to identify electrophysiological features driving classifier success. This approach combined elements of statistical inference and binary classification to investigate how neurophysiological features influenced the classification between different neural states. Such findings would provide insight into mechanisms facilitating the early phase of antidepressant response to stimulation and establish a putative predictive biomarker to guide treatment research.

## Materials and methods

### Patients

Eight patients (Table 1) were enrolled in a clinical trial of DBS in SCC for TRD (clinicaltrials.gov #NCT01984710). The inclusion and exclusion criteria for participation are identical to past DBS for depression protocols at Emory [9]. The primary outcome measure of clinical efficacy was the 17 item-Hamilton Depression Rating Scale (HDRS) [10]. The protocol was approved by the Emory University Institutional Review Board and the US Food and Drug Administration under an Investigational Device Exemption (G130107 held by H.S.M.), which was monitored by the Emory University Department of Psychiatry and Behavioral Sciences Data and Safety Monitoring Board and registered clinical trials.gov NCT01984710. Additionally, informed consent was obtained from each patient prior to the study starts.

**Table 1:** Sample characteristics.

Subject #	1	2	3	4	5	6	7	8	Mean (SD)
Gender	F	F	F	F	M	F	M	F	6 F/2 M
Age at OR (years)	43	43	66	58	53	56	37	44	50 (9.76)
Baseline HDRS-17 Score^a^	23.5	20.5	23.25	23.5	20.5	23.25	22.75	24.75	22.75 (1.50)
Post-OR HDRS-17 Score^b^	19	13	8	15	9	9	13	13	12.38 (3.66)

*F:* female, *M:* male, *SD:* standard deviation, *HDRS:* Hamilton Depression Rating Score.

^a^Average of 4 weeks before surgical procedures.

^b^A week after operating room stimulation.

### Tractography-guided DBS implantation

**Fig. 1:**
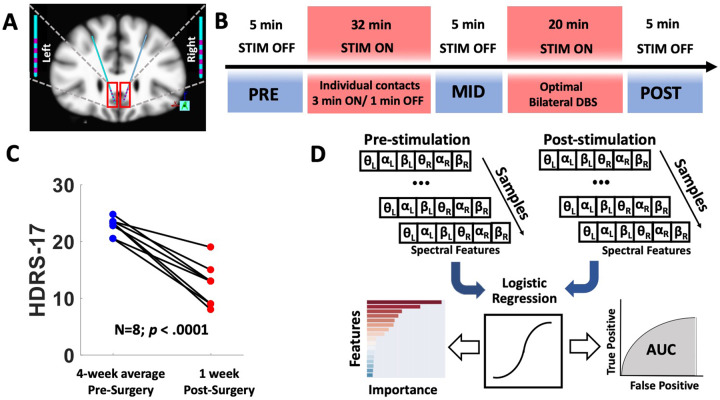
Experimental design and analytic procedures. **A** Schematic of bilateral leads implanted in the subcallosal cingulate. **B** Timeline of intraoperative procedures. Blue boxes (PRE, MID, POST) indicate epochs of SCC local field potentials (LFP) recording. **C** Decrease in symptom severity measured with Hamilton Depression Rating Scale (HDRS-17) following surgical implantation. **D** Machine learning (ML) analysis pipeline: classification models were developed from six LFP spectral features per subject to discriminate between PRE and POST epochs. ML outputs included area under the curve (AUC) and feature importance statistics.

Bilateral DBS leads (Medtronic model 3387 Minneapolis, MN), each with four contacts (model 3387; 1.5 mm inter-contact spacing), were implanted in the SCC (Fig. 1A). The surgical implantation procedure has been described in [9]. MR-diffusion image acquisition and registration procedures, as well as tractography processing, were conducted prior to surgery. Using these patient-specific tractography models, investigators identified the “optimal” target in each hemisphere as the convergence point of white matter fibers (forceps minor, cingulum bundle, frontostriatal fibers, and uncinate fasciculus) in the SCC region [9].

### Experimental design

As part of a parent study, experimental procedures were conducted in the operating room with the patient fully awake. First, stimulation was delivered in eight consecutive blocks (monopolar, unilateral stimulation with the following parameters: frequency = 130 Hz, current = 6 mA, pulse width = 90 μs, 3 min on, 1 min off) from each of the four contacts in each hemisphere. Following the initial stimulation protocol, four 5-min blocks of bilateral stimulation (identical parameters) were conducted, choosing in each hemisphere the tractography-defined “optimal” contacts (Fig. 1B). In total, the testing procedure was approximately 1 h in length. Local field potentials (LFPs) were simultaneously recorded from all eight contacts throughout intraoperative procedures. Light propofol anesthesia was administered for lead implantation. To ensure that patients were fully alert for the duration of the experimental procedures, anesthesia was discontinued for a minimum of 60 min prior to testing.

Data selected for analysis were 60 s of LFP recording at three time points. The first time point (PRE) was after surgical implantation of the electrodes with the patient fully awake and alert, but prior to any stimulation or experimental procedures. The second time point (MID) was taken midway through the testing procedure, approximately 1 h after the baseline recording and following unilateral stimulation at each contact. The third time point (POST) followed the period of extended exposure to bilateral stimulation at the “optimal” contacts (i.e., tractography-defined and confirmed with behavioral testing as in [11].

### Electrophysiological recordings

Intraoperative electrophysiological recordings were made using the NeuroOmega (AlphaOmega Engineering, Nazareth, Israel) and BlackRock (BlackRock Microsystems, Salt Lake City, UT) technologies. Bilateral electrodes implanted in the SCC region were connected to the recording system “headbox” via custom adaptors with a twist-lock connector. The custom adaptors parse each of the four contacts (per lead) into separate recording channels, allowing for simultaneous LFP recording from all contacts in both hemispheres. A grounding-pad was placed on the back of the shoulder, and recordings were referenced to the earlobe. Data were sampled at 44 kHz.

### Pre-processing of LFP data

All the analyses were performed using Matlab R2017a (The MathWorks Inc., Natick, MA, USA). The LFP recordings were down-sampled to 1000 Hz and 0.5–80 Hz bandpass filtered with zero-phase delay, and the DC offset was removed contact-wise by subtracting the average of each recording. Recording reference scheme was maintained instead of a local re-referencing scheme (e.g., bipolar pairs within the SCC) to capture variance across a broader swath of neural anatomy. In one patient, all recordings from the right hemisphere lead were removed from the analysis due to excessive noise. As in [12], LFP data were averaged across four contacts within each hemisphere. This procedure was selected to enhance the stability of inputs to the classifier, at the expense of a broader scope of investigation into location-specific effects within the SCC region (i.e., target engagement biomarkers). LFP signals were epoched into PRE, MID, and POST samples, each 60 s in length, and the signals were averaged within each hemisphere. Epochs were further divided into non-overlapping 1-s segments to account for non-stationarity in the data and then transformed into spectral power using a fast Fourier transform algorithm. Power spectral densities were binned as follows: *θ* (4–8 Hz), *α* (9–12 Hz), and *β* (13–30 Hz). Epoch length prevented the analysis of delta power (1–4 Hz). This procedure resulted in six spectral features per individual (three per hemisphere) at each analysis time point (Fig. 1D).

### Logistic regression with elastic-net regularization to classify LFP data

A multidimensional, data-driven classifier approach was used to assess for a statistical difference between electrophysiological recordings before and after extended intraoperative exposure to bilateral therapeutic stimulation. To test the hypothesis that the machine learning classification models can discriminate between the baseline (PRE) and the post-stimulation (POST) neural states, logistic regression classification models were trained with elastic-net regularization using the spectral features of the LFP recordings as the input vector (i.e., *θ*, 4–8 Hz; *α*, 9–12 Hz; *β*, 13–30 Hz from both hemispheres). Training the model involved identifying the optimal model parameters that define a projection from the multivariate feature space into the probability of each sample belonging to one of the two classes (PRE, POST). We used *K*-fold nested cross-validation (where *K* = 7 subjects) to fit the model parameters, optimize the hyperparameters [13], and evaluate the out-of-sample performance of the classification model. The average area under the receiver operating characteristic curve (AUC) across outer-loops was used to measure the separability between baseline and post-stimulation neural states. Figure 1D shows the analytic pipeline that we used for this analysis.

### Feature selection and analysis

We used an embedded shrinkage mechanism within elastic-net regularization to calculate the relative importance of the features to classifier success. This approach allowed us to identify the features that drove classifier success while simultaneously considering the contribution of all the features in the model. Elastic-net regularization forces the model fitting process to select only a subset of the input features that maximize the classification accuracy [14, 15]. Increasing a regularization parameter shrinks model coefficient values towards zero. This allows the less contributive features to have coefficient values close to or equal to zero. By calculating the cross-validation error as a function of the regularization parameter, a sparse subset of features that maximize the classification performance was identified (Supplemental Fig. 1). A relative importance measure for each feature was calculated based on the percentage of models in the inner-fold cross-validation in which the coefficient of the feature was greater than zero. Multiple comparisons on a one-way ANOVA test were used to find a subset of features whose relative importance was significantly larger than others (Supplemental Fig. 2) [16].

### Post hoc correlation with clinical outcomes

Change in symptom severity following intraoperative procedures was assessed within individuals as the mean weekly Hamilton Depression Rating Score (HDRS-17) of the 4 weeks prior to surgery, and the scores 1 week after surgery [10]. Means were compared using an rmANOVA. A post hoc analysis examined the correlation between observed percentage change in spectral features of LFP and percentage change in symptom severity (i.e., HDRS) 1 week following surgery. The statistical significance of Pearson’s linear correlation coefficient (*R*) was assessed with two-tailed *t*-tests of the null hypothesis that the slope of the regression equation is zero. Given the small sample size, a corrected *p* value threshold was determined using a false discovery rate procedure [16], which uses 5000 permutations of the data generating a distribution of *p* values.

### Post hoc comparison of classification success at three time points (PRE, POST, MID)

To investigate links between classification results and intraoperative procedures, we compared results of the primary analysis (PRE vs. POST) to an earlier time point (MID) that preceded an extended period of exposure to bilateral stimulation at the tractography-defined “optimal” contact. For completeness, a classifier comparing MID to POST samples was also constructed and importance scores, weighted by classifier success, were plotted for comparison with primary results (Supplemental Fig. 3). Additional subject-specific classifier models were examined to assess whether observed brain changes followed the optimal stimulation procedure specifically. To accomplish this, logistic regression models with elastic-net regularization and K-fold nested cross-validation compared LFP epochs recorded in the baseline (PRE) to recordings that followed exposure to non-specific stimulation (MID) and PRE to POST for any individual. AUC statistics were then submitted to a repeated measures analysis of variance to assess for mean differences in PRE-MID and PRE-POST classifier success.

## Results

### Antidepressant effects of intraoperative stimulation

Mean HDRS-17 in the eight patients declined from 22.75 ± 1.50 to 12.38 ± 3.66 in the week after surgery (Fig. 1C, 45.6% total decline). Three of the patients would have been considered “responders” to treatment by having decreased in their depression scores by more than 50%. The only stimulation that participants received was limited to the intraoperative period and was a total of 52 min across all trials and, more specifically, of 20 min in the “optimal” tractography-defined targets. The protocol determines there is no stimulation delivered in the 4 weeks following surgery.

### Acute changes in brain electrophysiology following exposure to intraoperative SCC-DBS

**Fig. 2:**
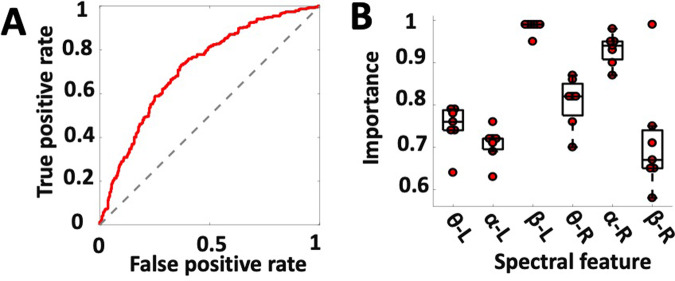
Acute changes in brain electrophysiology following exposure to intraoperative SCC-DBS. **A** Logistic regression classifier with elastic-net regularization discriminated between baseline (PRE) and post-stimulation (POST) LFPs in the SCC region, AUC_mean_ = 0.729; SD = 0.034, N = 7. **B** A feature importance score indicated the relative contribution of features to classifier success (PRE vs. POST).

Logistic regression classifiers with elastic-net regularization discriminated between baseline (PRE) and post-stimulation (POST) LFPs in the SCC region: area under the curve (AUC_mean_) = 0.729, SD = 0.034, *N* = 7. (Data from one patient was excluded from the analysis due to excessive noise in the right hemisphere lead.) The AUC in Fig. 2A represents the average out-of-sample classification performance; repeated iterations used data from six subjects for training the model and the remaining one subject for testing.

### Left beta power drives classifier success

**Fig. 3:**
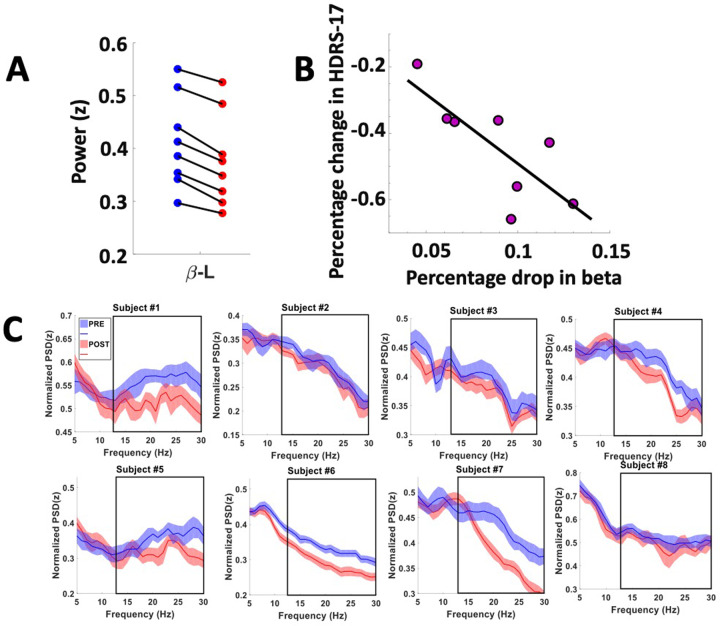
Decrease in beta power following intraoperative exposure to bilateral DBS at tractography-defined “optimal” contacts predicts early antidepressant effects. A Change in the left hemisphere beta power before (PRE: blue) and after (POST: red) exposure to DBS, *F(1,7)* = 96.23, *p*_corrected _< 0.001. **B** Left hemisphere LFP beta power decrease following intraoperative exposure to “optimal” DBS correlates with greater decrease in symptom severity 1 week following surgery, *r*(8) = 0.730, *p*_corrected_ = 0.04. HDRS Hamilton Depression Rating Scale. **C** Intraoperative SCC-LFP power spectral density (left hemisphere) in single subjects before exposure to DBS (PRE) and after (POST). Subject-level power spectral density (PRE: blue, POST: red). Black boxes indicate time window of beta power feature (13–30 Hz).

A feature importance analysis indicated the relative contribution of features to classifier success. Beta power (13–30 Hz), recorded from the left hemisphere SCC, outperformed all other features in the classification algorithm (Fig. 2B). To reduce the number of correlative comparisons in this small sample, we used importance scores to select two features for additional analysis. A post hoc comparison of mean spectral power (Fig. 3A) showed left SCC-LFP beta power reduced following stimulation to the tractography-defined “optimal” location in the SCC, *F*(1,7) = 96.23, *p*_corrected_ < 0.001, and right alpha power was increased relative to baseline, *F*(1,6) = 19.99, *p*_corrected_ < 0.01. Figure 3C shows the effect of this stimulation protocol on left beta power was observable in eight subjects.

### Intraoperative change in SCC beta power predicts early antidepressant response

Figure 1C shows symptom severity scores using the Hamilton Depression Rating Scale (HDRS-17) averaged 4 weeks before and a week after OR stimulation. Improved clinical outcome was observed across the sample following intraoperative procedures, *F*(1,7) = 58.25, *p*_corrected_ < 0.001. To investigate if changes in the LFP signal could predict the antidepressant response, we calculated the Pearson’s linear correlation between the percentage change of HRDS and the percentage change in left hemisphere beta power. We observed a statistically significant correlation between the decrease in left beta power and decrease in HDRS, *r*(8) = 0.730, *p*_corrected_ = 0.04 (Fig. 3B). However, the change in right alpha power and HDRS change did not show a significant correlation, *r*(7) = 0.476, *p*_corrected_ = 0.28. Features selection post hoc analyses was determined using importance scores and thus results do not negate the possibility that other features might also correlate with early changes in symptom severity.

### Failure to discriminate acute changes in brain electrophysiology following exposure to *non-optimal* DBS: implications for intraoperative protocol development

**Fig. 4:**
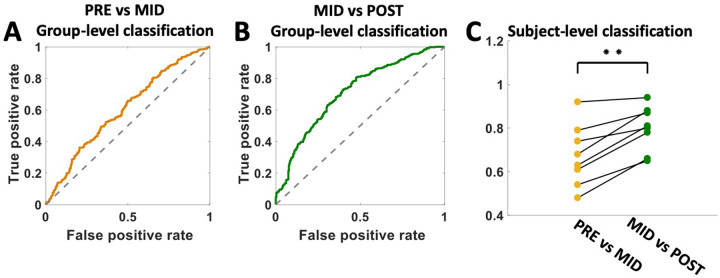
Classification analysis to specify the stimulation protocol to enhance acute intraoperative electrophysiological change. **A** Group-level logistic regression classifiers with elastic-net regularization failed to discriminate above chance between pre-stimulation baseline (PRE) and mid-procedure (MID) LFPs, AUC_mean_ = 0.574; SD = 0.04, N = 7 mid-procedure samples (MID) followed non-specific, unilateral testing at all contacts, including tractography-defined non-optimal contacts. **B** Group-level logistic regression classifiers reliably discriminated MID from post-stimulation (POST) LFP samples, where POST recording followed exposure to bilateral stimulation at the tractography-defined “optimal” contacts, AUC_MEAN_ = 0.684; SD = 0.059, *N* = 7. **C** Patient-level logistic regression classifier performance improved following exposure to tractography-defined “optimal” stimulation (*p* < 0.001).

Logistic regression classifiers with elastic-net regularization failed to discriminate above chance between baseline (PRE) and mid-procedure (MID) LFPs, AUC_mean_ = 0.574; SD = 0.04, *N* = 7 (Fig. 4A). Discrimination between mid-point (MID) and post-procedural (POST) recordings was above chance, AUC_MEAN_ = 0.684; SD = 0.059, *N* = 7 (Fig. 4B). Mid-procedure samples (MID) followed consecutive non-specific, *unilateral* testing at all contacts, including tractography-defined non-optimal contacts. Moreover, patient-level logistic regression classifier performance (Fig. 4C) improved following exposure to extended, bilateral stimulation at the tractography-defined “optimal” contact, *F*(1,7) = 28.04, *p* < 0.005.

## Discussion

Intraoperative exposure to stimulation delivered bilaterally in the tractography-defined targets generated electrophysiological changes in LFPs that were successfully discriminated by machine learning classifiers. More specifically, beta power in the left hemisphere emerged as a key driver of this classifier’s success, surviving the regularization penalty in all iterations of the cross-validation procedure. This decrease in beta power following intraoperative stimulation was observed within individuals, and the drop magnitude correlated with a post-operative decline in depressive symptom severity. These findings provide a putative physiological biomarker of brain-state changes that can be measured in vivo during DBS surgery and that can predict early antidepressant effects. From a clinical standpoint, the implementation of supplementary intraoperative bilateral stimulation may have facilitated a rapid-acting therapeutic response (causality not evaluated, here), in addition to benefits reported on the scale of weeks to months with chronic DBS.

Present results further clarify the observation that early therapeutic gains have become more pronounced across our consecutive study cohorts; the key difference being a combination of the precision of targeting and extended bilateral optimized stimulation in the operating milieu [7, 9]. This is evidence that opposes an explanation involving microlesion effects, which would be constant across study cohorts. The initial cohort of SCC DBS patients in our center did not receive systematic stimulation in the OR, and they had no antidepressant effects after surgery [17]. Though reports on treatment efficacy typically focus on long-term effects, uncontrolled observations in foundational SCC DBS studies state that daily post-operative exposure to stimulation resulted in significant symptom decrease 1 week later; symptoms return with the absence of stimulation over the ensuing 2 weeks [18]. Moderate reductions in symptoms have also been reported by other groups after 24 h of stimulation, compared to sham [19]. Taken together, these findings suggest that enhancement of a rapid and sustained antidepressant effect may be possible with exposure to DBS during the surgical procedures.

Moreover, present findings provide quantitative evidence to guide the implementation of an objective, intraoperative protocol. While we previously reported brain electrophysiological and behavioral changes following initial exposure to non-specific stimulation [20], these changes were not predictive of clinical outcomes. In this report, we present preliminary evidence that exposure to bilateral stimulation at the tractography-defined location induces brain-state changes that track with early treatment gains (Fig. 3). Beta power decrease in the left hemisphere SCC emerged as a putative biomarker predictive of the early antidepressant effect. Increased alpha power in SCC of the right hemisphere also contributed to classifier success on a group level, although it was not seen individually (Supplemental Fig. 4), and was not predictive of symptom change. A considerable literature—though yet to converge—links alpha power to depression [21–24], including a positive correlation between SCC LFP alpha power and symptom severity in TRD [25]. It should be noted that a causal association between intraoperative exposure to therapeutic stimulation is not established by these results, and future research will need to consider other change factors, such as time, subject fatigue, or social interaction in the intraoperative milieu.

Present findings establish a novel biomarker for future hypothesis testing. One priority is to investigate the anatomical specificity of the signal within the SCC region; present methods, such as reference scheme and use of averaging across contacts, privileged signal stability over location precision. Moreover, our treatment of consecutive samples as independent may have inflated statistical power. Another priority is to devise and test a model of treatment efficacy that accounts for these observed changes. It appears unlikely that the mechanism implicating SCC beta fluctuation after initial exposure to DBS involves suppressing primary pathology. However, suppression of pathological beta power is well established as a biomarker of DBS efficacy for treatment of Parkinson’s disease and is now under examination as an electrophysiological target of adaptive programming for movement disorders [7, 9, 12, 26, 27]. There is less evidence for pathological beta oscillations in depression. In fact, Clark and colleagues [12] observed an inverse relationship between pre-stimulation SCC beta power and pre-treatment symptom severity in TRD, particularly for somatic symptoms. Although not replicated in the present sample (Supplemental Fig. 5), perhaps due to a limited range of symptom scores, other groups have reported decreased pre-stimulation SCC LFP beta power during the negative emotional engagement that was associated with greater pre-treatment symptom severity [26, 28]. These authors speculate that SCC beta desynchronization is related to negative affective bias in TRD. Notably, the observed beta desynchronization was not predictive of symptom severity after 6 months of DBS treatment [26], suggesting a more complex interaction of treatment effects and SCC beta fluctuation at rest or during tasks. Neither acute nor chronic effects of SCC stimulation on these tasks induced beta changes were tested.

An alternative but complementary mechanistic hypothesis posits that beta power decrease observed following initial exposure to DBS is a necessary but insufficient brain change for full depression recovery, but one that facilitates or enhances adaptive capacity in a functional context. Though beta oscillations are classically associated with motor functions, a broader view considers a role for frontal beta power increase in the adaptive persistence of motor and cognitive states [29] diminished in depression [30]. Though speculative at present, it may be that rapid antidepressant effects with DBS reflect an adaptive beta response to task demands that is initially observed as a tonic power decrease in the resting state. Future research is needed to clarify SCC beta activity in TRD following acute and chronic exposure to DBS, perhaps related to psychomotor symptoms, which would not exclude a more generalized function in affective self-regulation.

The change in beta power is evident on the level of individual patients (Fig. 3C), and it may have been feasible to test the observed effects without the introduction of a classifier approach. However, a probabilistic, data-driven method allowed for simultaneous examination of multiple LFP features, and the model regularization reduced the probability of over-fitting in a small sample. Having identified a putative biomarker with a multivariate approach, priority for future research is to examine features beyond the scope of the current analysis (i.e., delta, gamma), as well as to clarify causal mechanisms implied by our interpretation of results. For example, it is unknown whether the early antidepressant response would covary with the duration or magnitude of intraoperative exposure to SCC DBS, and whether bilateral stimulation or the length of exposure to stimulation is driving the effect. Similarly, present methods cannot address with certainty the specificity of beta modulation to stimulation from the tractography-defined “optimal” location: extended, bilateral stimulation was given only at the optimal location used for long-term chronic stimulation, whereas non-optimal contacts were tested using unilateral settings for a shorter duration. Moderate doses of propofol for anesthesia have been previously associated with beta power changes. However, in our experiment, all anesthesia was discontinued more than 60 min prior to the initiation of any LFP recordings, and patients were awake and alert at the time of behavioral testing and recording. Also, no significant link was observed between MID vs. PRE beta change and early antidepressant response.

While present results instruct next steps for research into optimal target engagement and treatment mechanisms, the most urgent application is to investigate the predictive utility of the biomarker over the course of treatment. Our observation at present is that early gains are partially but not completely lost during a post-operative, 1-month wash-out period [7, 9]. It is yet unknown whether intraoperative stimulation-induced changes in beta power are predictive of eventual sustained clinical response to chronic therapeutic SCC DBS for TRD. To this point, chronic SCC DBS at the tractography-defined “optimal” locations resulted in a response rate of 88% (7 of 8) after 6 months of treatment (Supplemental Table 1). Ongoing studies are being conducted to examine LFP signatures and their relation to symptom improvement using chronic, ambulatory stimulation protocols and recording technologies embedded in the DBS pulse generator (Clinicaltrials.gov; NCT01984710). Our results point to SCC beta power as a putative biomarker of effective target engagement with SCC DBS in TRD with implications for tracking the DBS effects using chronic LFP recordings.

## Supplementary information

SUPPLEMENTAL MATERIAL

## Data Availability

The dataset will be archived at https://dabi.loni.usc.edu/explore as part of Project ID Project ID 1UH3NS103550, “Electrophysiological Biomarkers to Optimize DBS for Depression,” and can be made available for research purposes upon request to the corresponding author (HSM).

## Code availability

We freely provide the codes that have been used in this study upon a request made to the corresponding author (BM).
