# Supplementary material for: Intraoperative neural signals predict rapid antidepressant effects of deep brain stimulation
Source: Transl Psychiatry. 2021 Nov 3;11:551. doi: 10.1038/s41398-021-01669-0 (PMC8563808; doi:10.1038/s41398-021-01669-0)
Supplement: Supplementary file 1 — SUPPLEMENTAL MATERIAL [file 41398_2021_1669_MOESM1_ESM.docx]

**Supplemental Information**

**Intraoperative neural signals predict rapid antidepressant effects of deep brain stimulation**

Mohammad S E Sendi, Allison C Waters, Vineet Tiruvadi, Patricio Riva-Posse, Andrea Crowell, Faical Isbaine , John T Gale, Ki Sueng Choi, Robert E Gross, Helen Mayberg, Babak Mahmoudi

**Supplemental Table 1. Patient’s clinical information**

| **Subject #** | **1** | **2** | **3** | **4** | **5** | **6** | **7** | **8** | mean (SD) |
| --- | --- | --- | --- | --- | --- | --- | --- | --- | --- |
| **6-month HDRS-17 Score*** | 3 | 8 | 15 | 7 | 10 | 10 | 4 | 4 | 7.62  (4.03) |
| **Duration of Current Episode, (months)** | 24 | 7 | 36 | 36 | 24 | 120 | 60 | 13 | 40 (36.17) |
| **No. of Depressive Episodes (Life)** | 4 | 2 | 4 | 4 | 3 | 5 | 3 | 4 | 3.62 (0.91) |

*6-month after chronic stimulation at the optimized contacts.

SD=Standard deviation, HDRS: Hamilton Depression Rating Score

**Supplementary Figures**

**Supplemental Figure 1. The procedure for estimating the feature importance based on multi-variate classification and the elastic-net regularization.** A) The optimal regularization parameter was identified based on the minimum cross-validation error. B) increasing the regularization parameter forces the model parameters that are associated with the non-discriminative features to shrink and get values equal or close to zero. The features corresponding to non-zero model parameters that minimized cross-validation error were selected as the most important features.

**A**


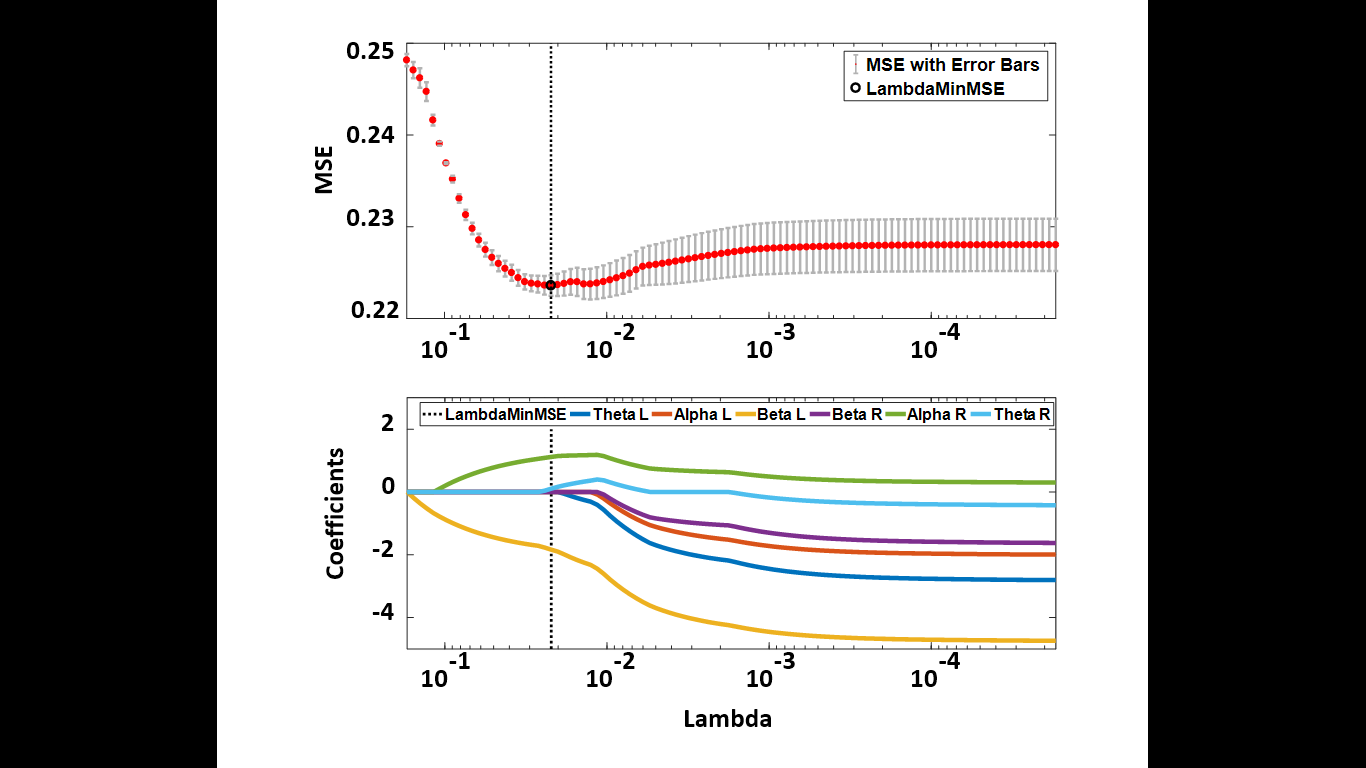


**B**

**Supplemental Figure 2.** **The statistical significance of the feature importance was calculated based on the frequency of each feature being identified as discriminative for all the patients** A) The relative number of occurrences of the biomarkers was compared using one-way analysis of variance (ANOVA) which found that the biomarkers was a statistically significant predictor of feature importance (p= 1.67e^-10^). B & C) Multiple comparison result shows that the number of occurrences for β_L and α_R are equally and significantly higher than the other predictors. D) Pair-wise comparison p-value. The difference between the number of occurrences for β_L and α_R is significant with the other predictor. The number of occurrences of β_L and the number of occurrences of α_R is not significantly different.


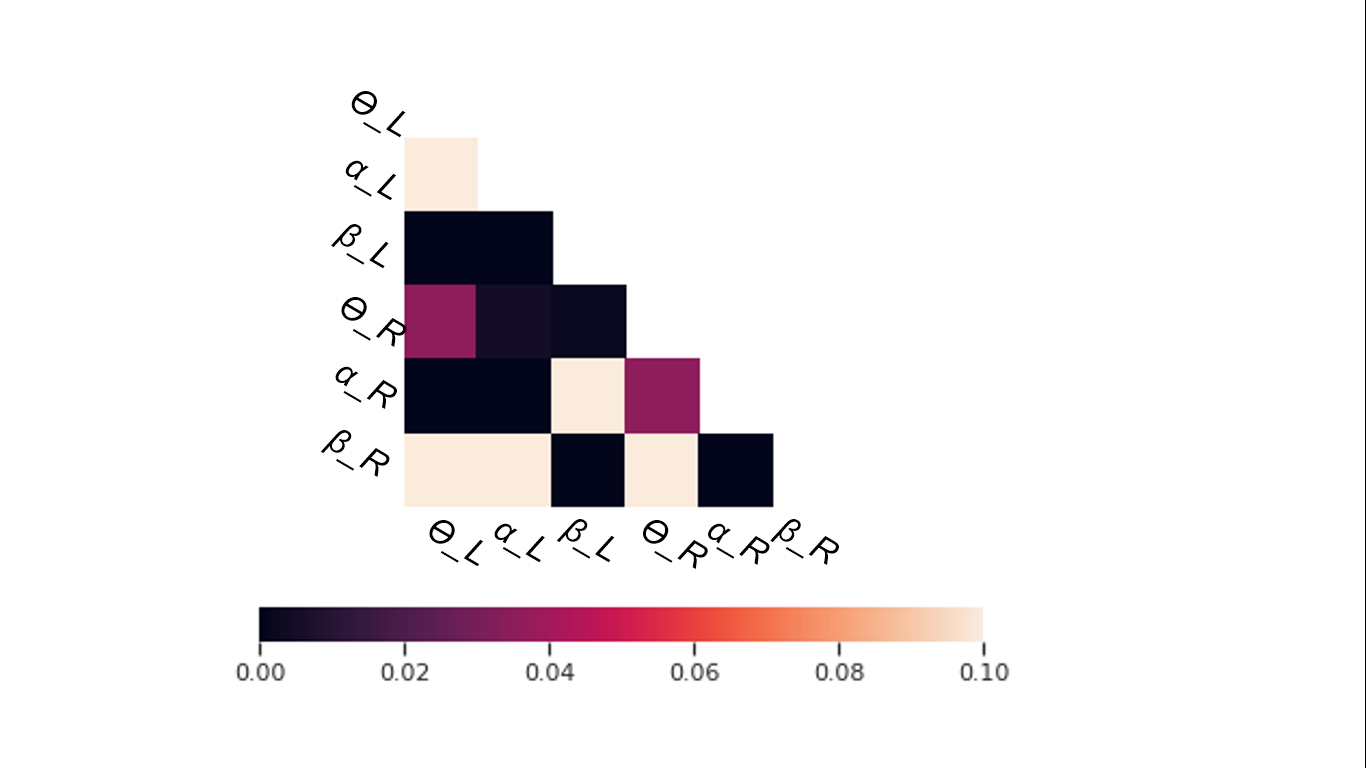


**B**

**C**

**A**

**D**

**Supplemental Figure 3. Feature Importance Scores Weighted by Classifier Success.** To investigate links between classification results and intraoperative procedures, we compared results of the primary analysis (PRE vs. POST) to an earlier timepoint (MID) that preceded an extended period of exposure to bilateral stimulation at the tractography-defined “optimal” contact. For completeness, a classifier comparing MID to POST samples was also constructed and importance scores, weighted by classifier success, were plotted for comparison with primary results. Within each fold of 7-fold cross-validation (where K=7 patients), we multiplied the classification AUC (described in *Logistic regression with elastic-net regularization to classify LFP data***)** by the feature importance values (described in *Feature selection and analysis*). A) Comparing the weighted feature importance of the PRE vs MID with PRE vs POST classification. Beta left and alpha right of the latter classifier are relatively more important that other features. B) Comparing the weighted feature importance of the MID vs POST with PRE vs POST classification. Beta left and alpha right of the latter classifier are relatively more important that other features.


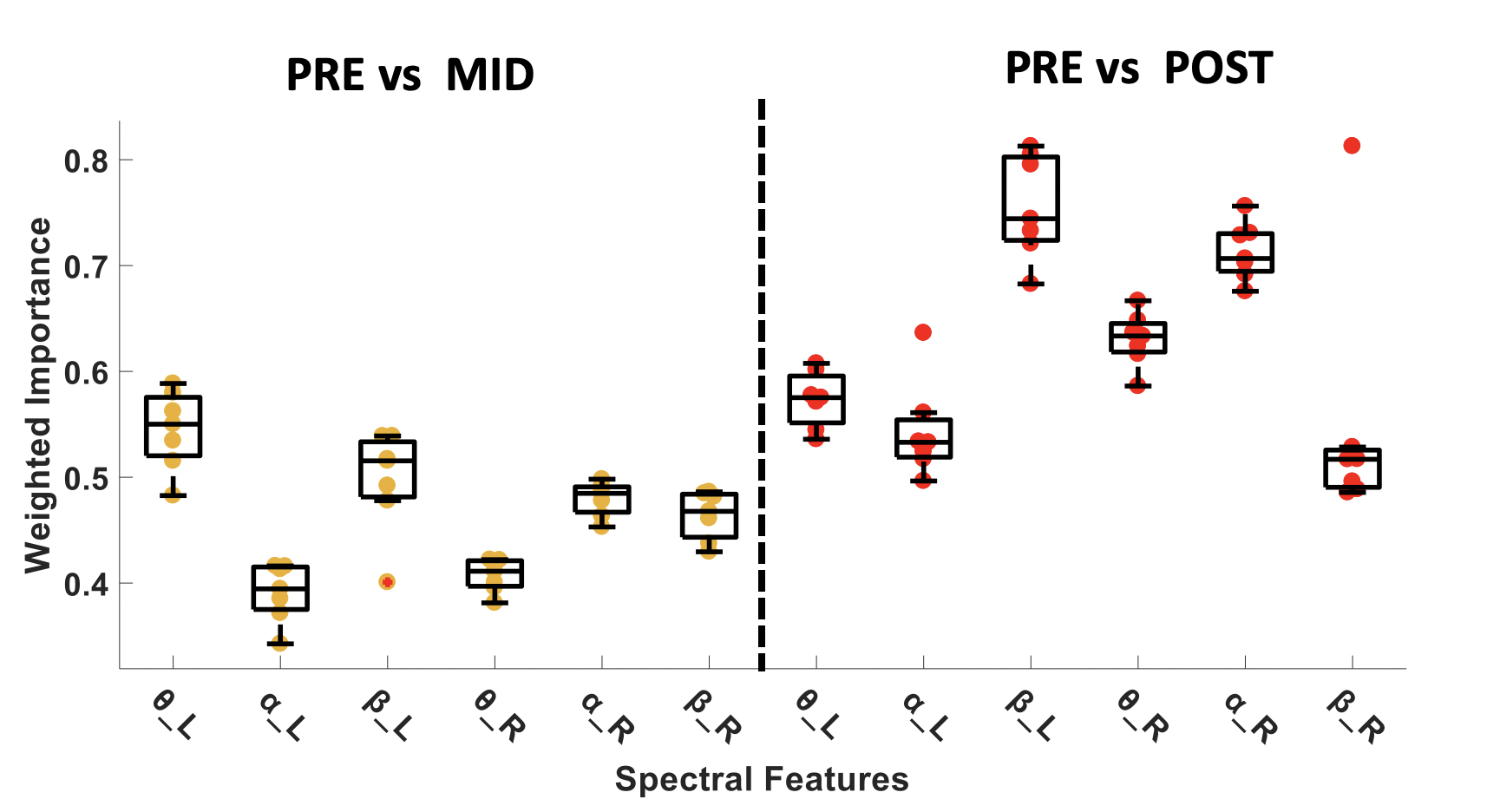

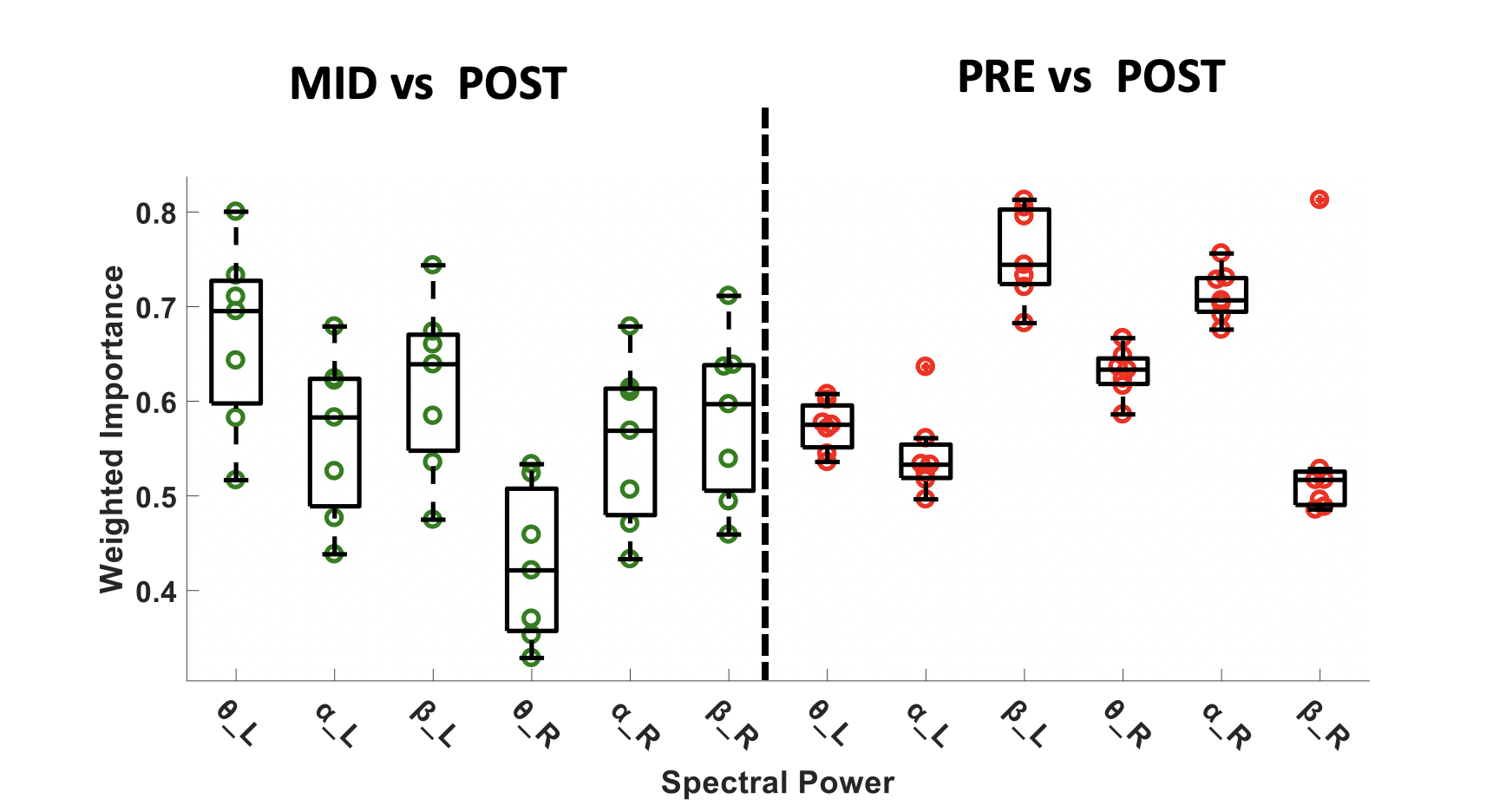


**A**

**B**

**Supplemental Figure 4**. **Increased right alpha power following intraoperative exposure to bilateral DBS at tractography-defined “optimal” contacts.** Subject-level power spectral density (PRE: blue, POST: red) recorded in right hemisphere subcallosal cingulate. Logistic regression classifiers with elastic net regularization discriminated between baseline (PRE) and post-stimulation (POST) local field potentials (LFPs) in the SCC region: area under the curve (AUC_MEAN_) = 0.729, SD = 0.034, N=7. Both left hemisphere beta power (see Figure 2) and right hemisphere alpha power (below) were the most important features that contributed to classifier success.

**Supplemental Figure 5. Electrophysiological correlates of pre-treatment symptom severity in the stimulation naïve brain.** Baseline beta did not predict symptom severity in this sample, *r*(8) = 0.419, *p* = 0.30, an association previously reported by Clark et al., 2016^1^. Outcome discrepancy might be explained by methodological and analytic differences between inquiries. Unlike Clark and colleagues, for example, our baseline severity scores (an averaged across 4 weeks preceding surgical implantation) showed insufficient variance for a correlation analysis. Moreover, the location of stimulation in the SCC region may have varied, see^2^.

**References:**

1 Clark DL, Brown EC, Ramasubbu R, Kiss ZHT. Intrinsic Local Beta Oscillations in the Subgenual Cingulate Relate to Depressive Symptoms in Treatment-Resistant Depression. *Biological Psychiatry* 2016; **80**: e93–e94.

2 Clark DL *et al.* Tract-based analysis of target engagement by subcallosal cingulate deep brain stimulation for treatment resistant depression. *Brain Stimulation* 2020. doi:10.1016/j.brs.2020.03.006.
